# Pars plana vitrectomy under melphalan irrigation for recurrent retinal detachment in eyes treated for retinoblastoma: a case report

**DOI:** 10.1186/s12886-020-1315-7

**Published:** 2020-01-28

**Authors:** Christina Stathopoulos, Jessica Sergenti, Marie-Claire Gaillard, Francis L. Munier, Alejandra Daruich

**Affiliations:** 1 Department of ophthalmology, University of Lausanne, Jules-Gonin Eye Hospital, Fondation Asile des Aveugles, Lausanne, Switzerland; 2 Ophthalmology Department, Necker-Enfants Malades University Hospital, APHP, Paris Descartes University, 149 rue de Sèvres, 75015 Paris, France

**Keywords:** Retinoblastoma, Pars plana vitrectomy, tractional retinal detachment, melphalan irrigation

## Abstract

**Background:**

Tractional retinal detachment with or without secondary tear is a rare complication reported in less than 0.5% of in eyes treated for retinoblastoma. Pars plana vitrectomy (PPV) in eyes with history of retinoblastoma has been associated with a significant risk for recurrence, extraocular spread, and systemic metastases. We report here the successful management by PPV under melphalan irrigation of 2 children presenting with tractional retinal detachment after retinoblastoma therapy and scleral buckle surgery.

**Case presentation:**

A 7-year-old girl with a history of bilateral retinoblastoma (group D) presented with light perception best-corrected visual acuity (BCVA) and tractional retinal detachment (RD) in her left eye, 3 years after the last intra-arterial chemotherapy (IAC) injection. Moreover, she had history of left eye rhegmatogenous RD treated by scleral buckle 1 month after the last IAC and cataract surgery 12 months later. PPV associated with retinectomy, laser photocoagulation and silicone oil tamponade was performed. Silicone oil was removed 4 months later. Fifteen months after PPV, BCVA had increased to 20/32 without recurrence of RD and no evidence of tumor activity. A 7-year-old boy with a history of unilateral retinoblastoma (group D) in his left eye presented with rhegmatogenous RD 21 months after the last treatment for retinoblastoma. Scleral buckle surgery was performed, but 3 weeks later the patient presented with tractional RD associated with proliferative vitreo-retinopathy. BCVA was counting fingers. PPV associated with membrane peel, laser photocoagulation and silicone oil tamponade was performed. Silicone oil was removed after 5 months followed by cataract surgery 5 months later. Twenty months after PPV, BCVA was 20/20 and there was no sign of tumor recurrence.

**Conclusions:**

PPV under melphalan irrigation, with retinectomy, if necessary, and silicone oil tamponade, allows anatomical and functional improvement in eyes with history of retinoblastoma and scleral buckling developing tractional RD.

## Background

Serous retinal detachment (RD) is a frequent finding in eyes with retinoblastoma at presentation, commonly caused by tumor-related exudation and usually completely resolving after tumor regression. Transient spontaneously resolving RD can also follow or worsen after rapid tumor regression. In some instances, however, eye-preserving retinoblastoma management can be complicated by secondary rhegmatogenous and/or tractional RD. Rhegmatogenous RD has been reported in 1% [1, 2] to 6% [3, 4] of the cases, mostly due to atrophic retinal holes. Tractional retinal detachment with or without secondary tear remains a rare complication reported in less than 0.5% of cases [1]. Left untreated, such detachments may compromise further tumor control and/or vision and globe preservation.

Intraocular surgery in the context of retinoblastoma is feared because of the risk of tumor spread. Secondary RD in eyes with retinoblastoma are preferentially managed with an external approach, as scleral buckle surgery [5–7] or enucleation [8]. Pars plana vitrectomy has been associated with a significant risk for tumor relapse, extraocular spread and systemic metastases [9], and thus reserved for selected cases with no other alternative for restoring visual function [10]. Although no consensus exists, usually a 6 to 18 month disease-free follow-up is preferred before considering scleral buckling with external drainage or vitrectomy [1, 2, 11].. Management of recurrent RD in retinoblastoma eyes has not been further reported.

We report here the successful management of tractional retinal detachment with PPV under melphalan irrigation in 2 children treated for retinoblastoma and with previous scleral buckling surgery.

## Case presentation

### Case 1

**Fig. 1:**
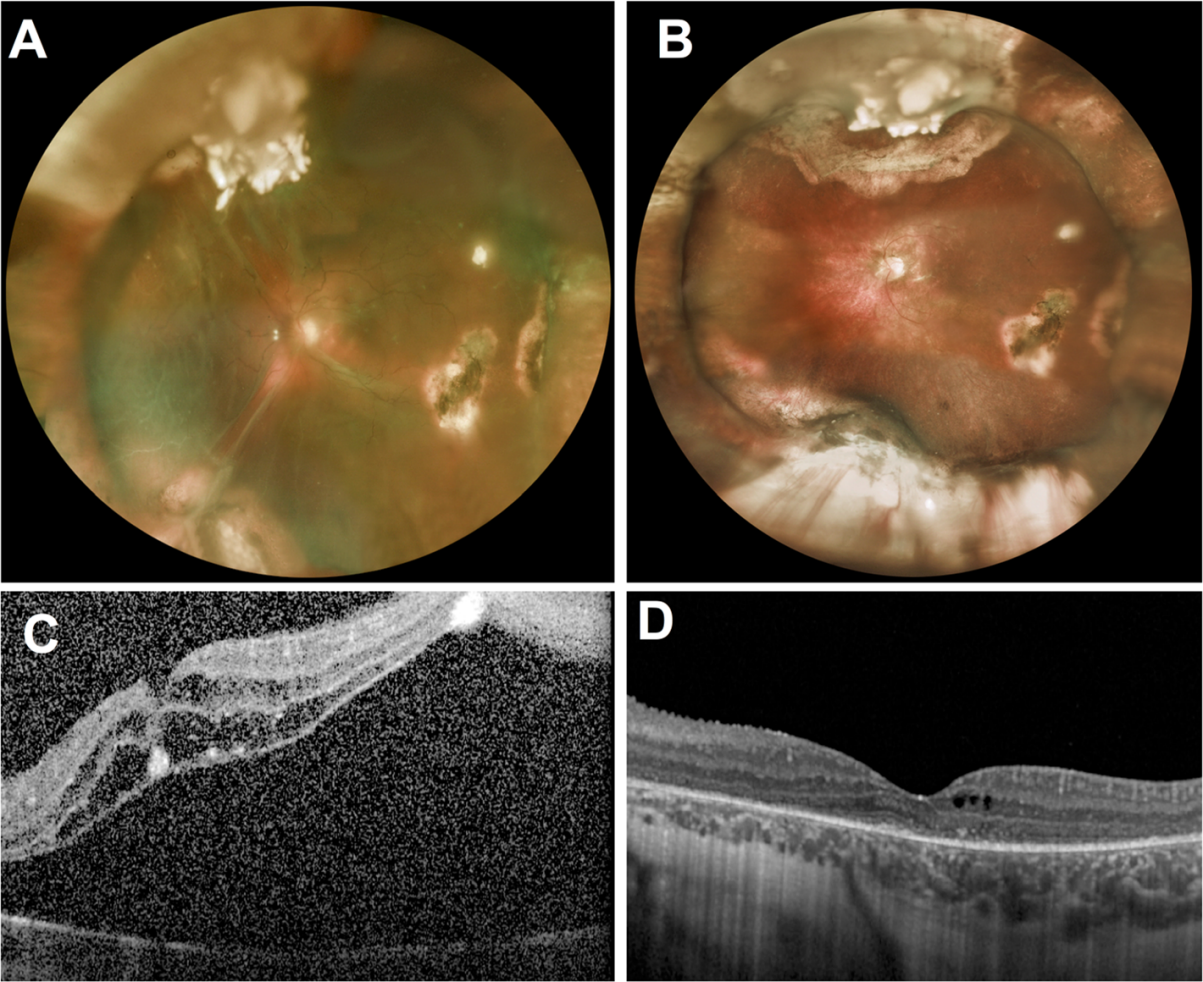
**a-c**: Fundus photograph and optical coherence tomography of a 7-year-old girl with history of retinoblastoma and scleral buckle surgery for RRD, presenting 3.4 years later with total tractional retinal detachment in her left eye. BCVA was light perception. **b-d**: Fundus photograph and optical coherence tomography of the same patient 12 months after pars plana vitrectomy under melphalan irrigation, retinectomy, laser and silicone oil tamponade for 4 months. BCVA increased to 20/40

A 4-year-old girl with bilateral International Intraocular Retinoblastoma Classification (IIRC) [12] group D retinoblastoma diagnosed at the age of 20 months was treated with intraarterial injections of combined melphalan and topotecan for a relapse in the left eye. Previous treatments of that eye included 2 cycles of systemic chemotherapy (carboplatin, etoposide), 2 intraarterial and 10 intravitreal melphalan injections as well as numerous focal treatments (cryotherapy and thermotherapy). After the third intraarterial injection, she developed a total rhegmatogenous RD secondary to the formation of an atrophic hole at the basis of the regressed relapse. Scleral buckling without drainage but with an intraoperative anterior chamber tap to allow a sufficient tightening of the band was done, resulting in a complete reattachment of the retina. One year later, she developed a cataract impeding the fundus view and underwent lens aspiration with posterior capsulorhexis, anterior vitrectomy and intracapsular lens implantation. Two and a half years after cataract surgery, at the age of 7, vision in that eye suddenly decreased to light perception. Fundus examination showed a total macula-off tractional retinal detachment with no identifiable retinal break (Fig. 1 a and c). PPV under melphalan perfusion (5 μg/ml) was performed. The exclusively tractional nature of the detachment originating from retinal dragging by the tumor scar was confirmed intraoperatively, since no retinal break was identified, and subretinal fluid could not be evacuated after perfluorocarbon liquid injection. To allow the retina to re-applicate, a retinectomy was created around the tumor scar, followed by laser photocoagulation and silicone oil tamponade. Cytologic examination of the vitrectomy fluid did not find any malignant cells. Silicone oil was removed 4 months later. At a 15-month post vitrectomy follow-up, the child had remained relapse- and metastasis-free without recurrence of RD and a best-corrected visual acuity (BCVA) of 20/32 in the vitrectomized eye (Fig. 1 b and d).

### Case 2

**Fig. 2:**
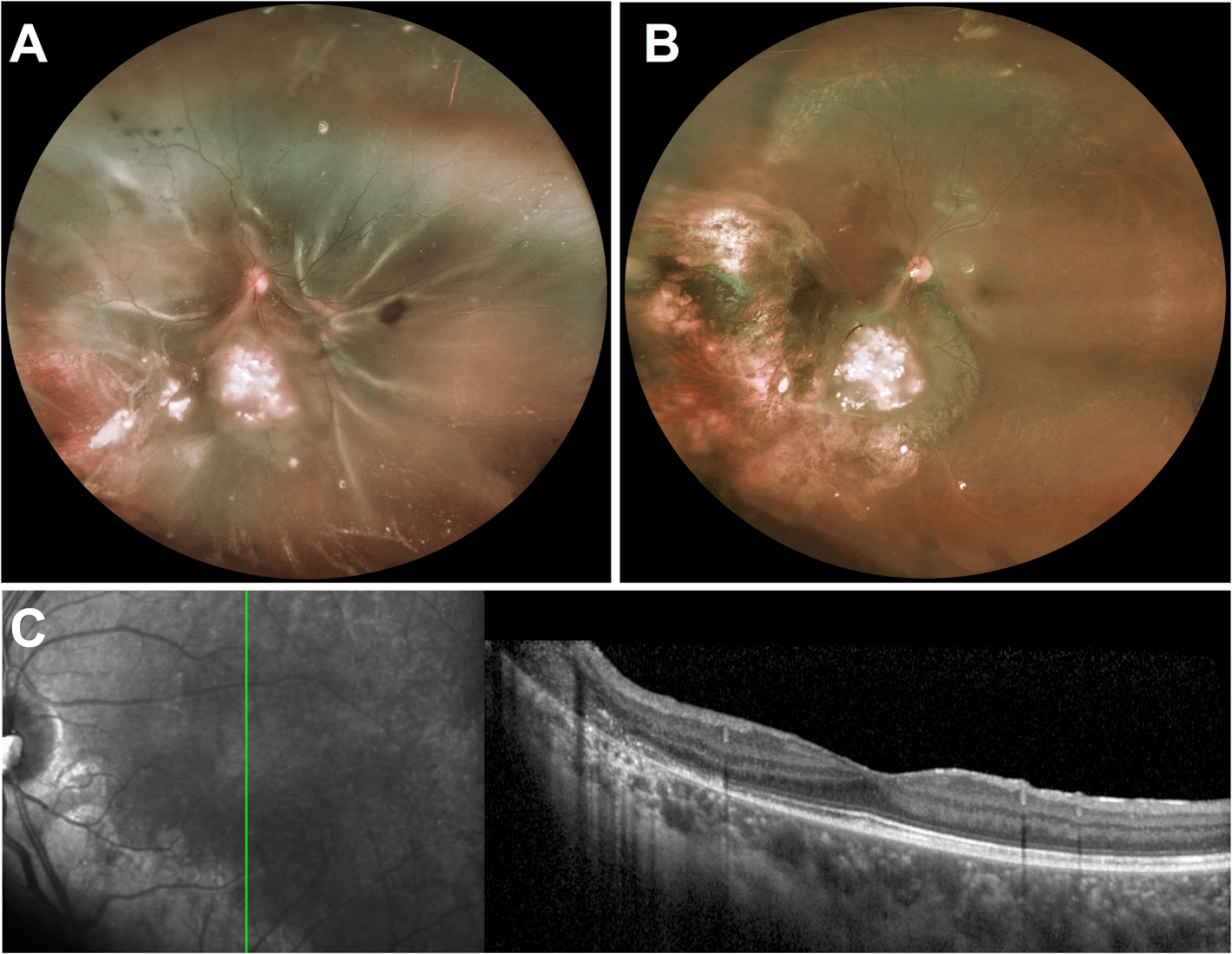
**a**: Fundus photograph of a 7-year-old boy with history of retinoblastoma presenting with total tractional retinal detachment and proliferative vitreo-retinopathy in his left eye after scleral buckle surgery performed 21 months after the last tumor treatment. BCVA was counting fingers. **b-c**: Fundus photograph and optical coherence tomography of the same patients 14 months after pars plana vitrectomy under melphalan irrigation, membrane peel, laser photocoagulation and silicone oil tamponade for 5 months. BCVA increased to 20/32

A boy diagnosed with unilateral IIRC group D retinoblastoma at the age of 5 was successfully treated with 3 cycles of intravenous chemotherapy (carboplatin, etoposide) as well as 8 intravitreal and 6 intracameral melphalan injections. At the age of 7, 21 months after the last tumor treatment, he presented with sudden vision decrease in the affected left eye. BCVA was 20/250. Fundus examination showed a total macula-off retinal detachment due to a large tear next to the tumor scar. Scleral buckling without drainage was performed but the retina failed to reapplicate despite appropriate indentation of the buckle, as proliferative vitreoretinopathy (PVR) developed three weeks later, particularly involving the macula (Fig. 2a) and inducing tractional retinal detachment. BCVA decreased to counting fingers. He underwent PPV under melphalan (5 μg/ml) perfusion with membrane peel, additional laser photocoagulation around the retinal tear and silicone oil tamponade. Cytologic examination of vitrectomy fluid was negative for malignant cells. Silicone oil removal was performed 5 months later, and cataract surgery with posterior capsulorhexis and posterior chamber intraocular lens implantation was performed 10 months later. At 20-month post vitrectomy follow-up, the child had remained relapse- and metastasis-free, with no recurrence of retinal detachment and a BCVA of 20/20 (Fig. 2 b, c).

## Discussion and conclusions

We described here 2 unusual cases of tractional retinal detachment after retinoblastoma treatment, and scleral buckle surgery for RRD, that were successfully managed by PPV under melphalan irrigation.

Secondary RD occurring during treatment of retinoblastoma is to date a rarely reported complication but this may change in the future as more advanced cases are now tried to be salvaged. Rhegmatogenous RD due to an atrophic hole/or a horsehoe tear have been reported after different treatment modalities including intravenous chemotherapy, external beam radiotherapy or plaque brachytherapy with the role of focal treatments, as laser photocoagulation or cryotherapy, being emphasized [1, 2, 13]. Recently, a greater prevalence of this complication was found in eyes treated with intraarterial chemotherapy with or without focal treatments compared to those treated with intravenous chemotherapy and/or external irradiation (6% versus 1%) [3] attributed by the authors to the rapid tumor regression and the atrophic hole formation at the basis of the tumor necrosis after intraarterial chemotherapy. RD of tractional origin occur even less frequently, and have only been reported in a few cases [1, 10].

To date there is no consensus on the best technique nor the acceptable disease-free interval to manage secondary RD in cases of retinoblastoma. First concern should be to rule out an active tumor as part of the detachment underlying mechanism or as concomitant feature at time of the complication diagnosis. Whereas scleral buckling is an appropriate option for rhegmatogenous RD [2, 14] extensive tractional RD can only be successfully managed with intraocular procedure [1, 2, 6, 10]. We reported here 2 cases of tractional RD successfully managed by PPV. In the first case, tractional forces exerted by the tumor scarring process induced retinal pulling and detachment without evidence of retinal break. A large retinectomy performed around the tumor scar allowed retinal reapplication. Retinectomy has been previously reported in two retinoblastoma eyes [14] with successful anatomical results. However, retinectomy in children should be restricted to cases with no other option for retinal reapplication, such as Case 1, where traction from the tumor scar induced a recurrence of RD after previous scleral buckling procedure. The second patient presented first with a rhegmatogenous RD that was also treated by scleral buckle surgery. PPV was performed due to the development of PVR affecting the macula. However, the large retinal break localized at the tumor edges, makes us suppose that it could be secondary to the tractional forces exerted by the tumor scarring process inducing retinal pulling, as in the first case, but more severe resulting in a secondary tear. Retinal breaks often occur at the edge of hard calcified retinoblastoma scars, resulting in an irregular shape and hardness of the break edge, that could be difficult to treat with scleral buckling without combined vitrectomy [15], especially if extensive PVR is already present [6]. Additionally, PPV was performed under melphalan perfusion (5 μg/ml), to increase the safety of the procedure (by preventing a potential active tumor spread through the scleral ports and/or the choroid), at a dose that was proved non-toxic for the retina in a rabbit model and in 3 retinoblastoma patients [14, 16, 17].

Recovery of visual acuity was seen in both patients, even though spectral-domain optical coherence tomography of the macula at the last follow-up showed a slightly atrophic fovea with alterations on photoreceptor and retinal pigment epithelium layers in Case 1.

In conclusion, pars plana vitrectomy under melphalan irrigation, with retinectomy, if necessary, and silicone oil tamponade is a reasonable treatment option in eyes with controlled retinoblastoma developing tractional RD after rhegmatogenous RD treated with scleral buckle.

## Data Availability

The data of this case report are available from the corresponding author on reasonable request.

## Abbreviations

BCVA: Best corrected visual acuity
IAC: Intra-arterial chemotherapy
IIRC: International Intraocular Retinoblastoma Classification
PPV: Pars plana vitrectomy
PVR: Proliferative vitreo-retinopathy
RD: Retinal detachment
